# The Role of Specular Reflections and Illumination in the Perception of Thickness in Solid Transparent Objects

**DOI:** 10.3389/fpsyg.2022.766056

**Published:** 2022-02-17

**Authors:** Masakazu Ohara, Juno Kim, Kowa Koida

**Affiliations:** ^1^Department of Computer Science and Engineering, Toyohashi University of Technology, Toyohashi, Japan; ^2^School of Optometry and Vision Science, University of New South Wales, Sydney, NSW, Australia; ^3^Electronics-Inspired Interdisciplinary Research Institute, Toyohashi University of Technology, Toyohashi, Japan

**Keywords:** 3D perception, motion, shape, surface material, specular reflection, refractive distortion, transparency

## Abstract

Specular reflections and refractive distortions are complex image properties of solid transparent objects, but despite this complexity, we readily perceive the 3D shapes of these objects (e.g., glass and clear plastic). We have found in past work that relevant sources of scene complexity have differential effects on 3D shape perception, with specular reflections increasing perceived thickness, and refractive distortions decreasing perceived thickness. In an object with both elements, such as glass, the two optical properties may complement each other to support reliable perception of 3D shape. We investigated the relative dominance of specular reflection and refractive distortions in the perception of shape. Surprisingly, the ratio of specular reflection to refractive component was almost equal to that of ordinary glass and ice, which promote correct percepts of 3D shape. The results were also explained by the variance in local RMS contrast in stimulus images but may depend on overall luminance and contrast of the surrounding light field.

## Introduction

Images result from the complex interplay between illumination and a surface's material composition and three-dimensional (3D) shape. Although we have vivid experiences of surfaces with material properties (e.g., glossy, matte, opaque, or transparent), observers are often biased in their report of either a surface's material appearance or 3D shape (Nishida and Shinya, 1998; Belhumeur et al., 1999; Nefs et al., 2006; Khang et al., 2007; Vangorp et al., 2007; Wijntjes and Pont, 2010; Mooney and Anderson, 2014; Dövencioglu et al., 2015; Marlow et al., 2015; Todd et al., 2015). For example, previous work has shown that the perceived 3D shape of opaque objects tends to be underestimated (Koenderink and van Doorn, 1992; De Haan et al., 1995; Todd et al., 2004; Bernhard et al., 2016). However, some researchers have shown the opposite pattern of results can occur (Mooney and Anderson, 2014). Adding sharp specular reflections was found to increase perceived convexity in excess of the surface's true curvature (Mooney and Anderson, 2014). In other words, glossier surfaces tend to be perceived as bumpier than diffuse surfaces.

Another surface property that influences perceived shape is the refractive nature of transparent objects. Images of thick transparent objects have complex structure attributed to their refractive power, shape and material composition (Fleming et al., 2011; Schlüter and Faul, 2014). It is also complex because natural transparent objects tend to reflect specular reflections off their surfaces. How do the refractive and reflective properties of transparent surfaces influence the perception of their 3D shape?

Although perceptual judgments of transparency loosely correspond to their refractive index (RI) (Fleming et al., 2011), human observers are not able to accurately estimate the RI of transparent objects. Researchers proposed that perception of transparency was estimated based on the background distortion seen through transparent objects (Fleming et al., 2011; Fleming, 2014; Todd and Norman, 2019). Another study proposed that background distortions alone are not sufficient for perceiving RI because they depend on both the shape and distance of the object from the background (Schlüter and Faul, 2014). Rather than observers matching internal experiences of refractivity, these researchers found that observers tended to match surfaces directly based on similarity in image cues: specular reflections and the distortion field.

**Figure 1:**
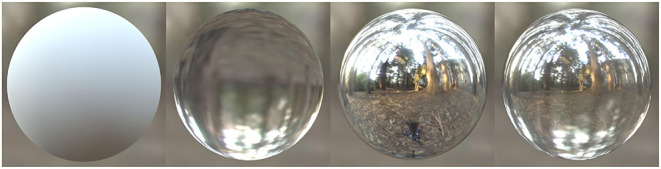
Object surface property affects perceptual thickness. Smooth spheres with different material properties are shown: diffuse, transparent, specular, and transparent + specular (1:1) from left to right. The transparent object appears flatter than the other objects, while the specular object and transparent + specular (1:1) object look thicker than the other objects.

Further studies have used gauge figure tasks to estimate variations in perceived surface slant (i.e., surface curvature) and found that the 3D shape of objects with semi-opaque reflectance properties tends to be perceptually underestimated (Chowdhury et al., 2017; Schlüter and Faul, 2019). These interactions between perceived shape and material properties suggest that the perception of both shape and materials depends on similar sources of image-based information. Previously, we systematically varied the simulated material composition of objects from refractive to reflective, with different amounts of specular reflectance. We found that the thickness of objects with specular reflectance tends to be perceptually overestimated, and thickness of transparent objects tends to be perceptually underestimated. We also found that the objects with 50% specular and 50% transparent components were perceived to have similar thickness to that of 100% specular surfaces, as demonstrated in Figure 1 (Ohara et al., 2020). These results indicate that the specular component can dominate in the perception of 3D shape. This finding raises questions about what specular-refraction blend ratio would best mix these components to induce veridical perception of object thickness, and how perceived thickness alters as a function of the ratio of these two components. To address this question, we investigated thickness perception of objects that have different ratios of specular and refractive components. We also explored what image cues could determine this thickness perception.

## Materials and Methods

### Participants

Eight adult observers participated in an initial main experiment and nine observers participated in a follow-up experiment. All of whom had normal or corrected-to-normal vision. The participants ranged in age from 23 to 27 years. All participants were naïve to the purposes of this experiment. Informed consent was obtained from all participants. Procedures were approved by the Toyohashi University of Technology ethics committee. All research was performed in accordance with the relevant guidelines and regulations.

### Stimuli

**Figure 2:**
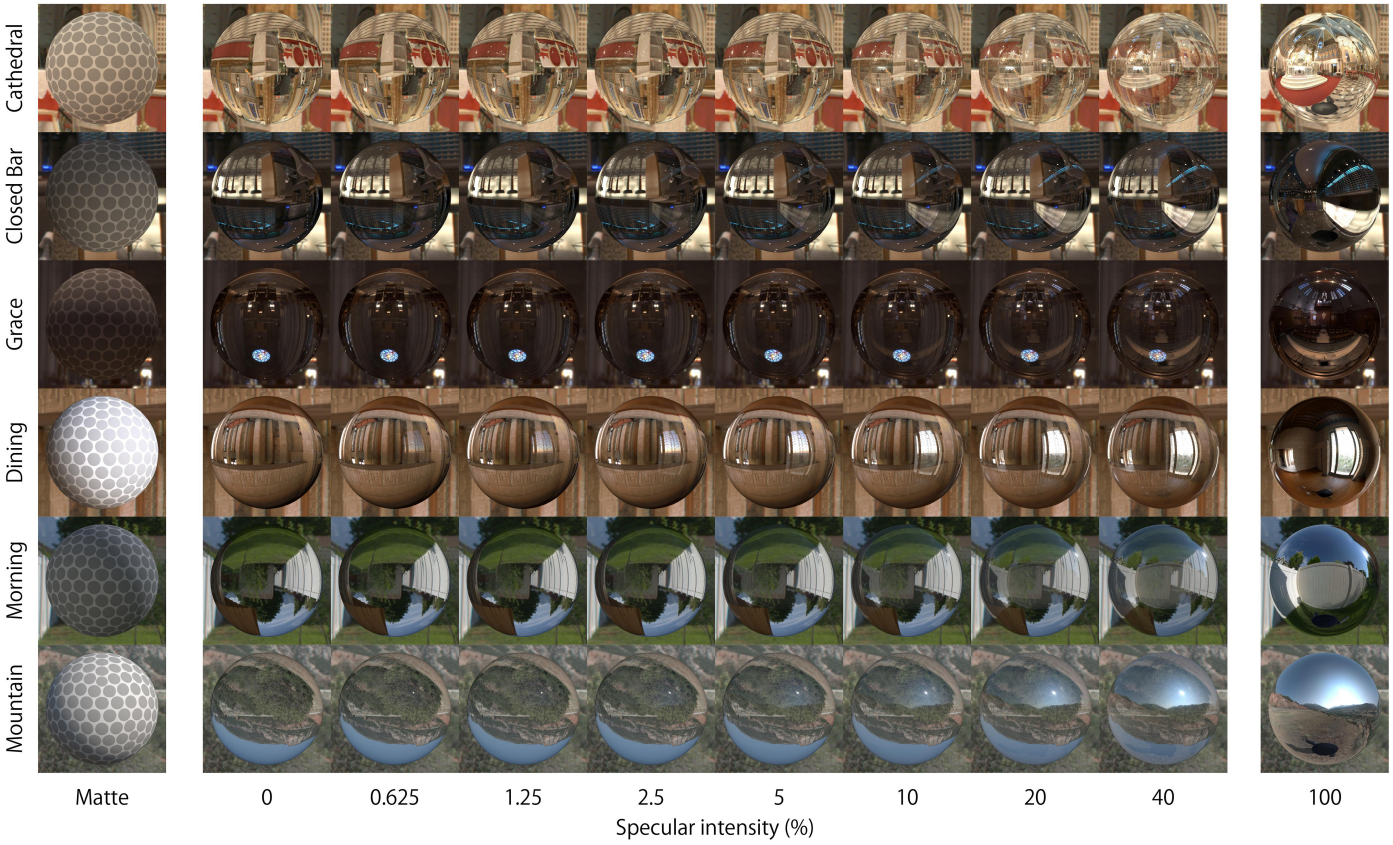
Appearance of stimuli. Matte and Test objects (specular ratio: 0–40%) were used in the experiment with six light probes. The 100% specular surface was not used in the experiment.

Figure 2 shows the objects used in this experiment. The shape of all samples and references was a sphere. The reason why we initially used only spherical shapes was because spheres were previously found to induce highly reproducible effects, compared with bumpy shapes used in our previous report (Ohara et al., 2020). There were two object stimuli: a reference and test stimulus. The reference was always a matte surface and was wrapped with a uniform dot texture (Figure 2, left). The test surface had either refractive, specular, or a mix of these components. Both refractive and specular components were generated separately, and then mixed by a weighted sum of them. There were eight ratios of purely specular component [0 (purely refractive), 0.625, 1.25, 2.5, 5, 10, 20, and 40%]. Although rendering transparency in this way is not physically correct, it held constant the refractive index at a value of 1.51. If physically correct rendering was applied to the transparent object, then changing the specular ratio would also alter the simulated refractive index and generate refractive distortions. However, we did not want the results to be solely driven by refractive distortions, since the aim in this study was to understand the relationship between the reflective properties of an object's surface and perceived thickness. The potential effect of this rendering on perceptual outcomes is considered further in the Discussion.

The reason why we did not use the objects with more than 40% specular reflectance in our experiments was because the experimental results for these objects can be predicted from our previous studies (Ohara et al., 2020). In our previous study, objects with 50% transparency and 50% specular reflectance were perceived as having similar thickness to the object with only specular reflectance. There might be an optical difference between the specular ratio of 40% (max ratio of our current experiment) to 50% (ratio used in previous experiment), but the perceptual difference could be small.

All the stimuli were rendered using the open-source rendering package Blender 3D (Ver. 2.78, https://www.blender.org/). The 3D geometry was created in Blender 3D by taking an initial Ico Sphere with 20,480 triangle faces, 10,242 vertices (i.e., subdivisions = 6). This object's diameter was 1 Blender Units (BU, corresponding display size: 4.7 deg). All the stimuli were rendered with Cycles Render in Blender 3D. Cycles Render is a physically based ray tracing render engine designed for high-quality lighting simulation and animation.

The purely refractive component was simulated using a Refraction BSDF shader with zero roughness and applying Fresnel equations using the Mix shader. The Fresnel shader reproduces changes in refractive distortion that depend on the angle of the incident light. The RI used for generating refractive images was 1.51, which corresponded to crown glass. The purely specular component was generated by a Glossy BSDF shader with zero roughness and without Fresnel shading. Supplementary Figure S1 shows the Blender node setup of this surface material.

We rendered images with limited linear tone mapping; the intensity of the specular highlights was limited by setting the exposure, and any values exceeding the dynamic range of our standard RGB rendering were set to a maximum of 255. The monitor's calibrated gamma for displaying these images was 2.2.

We used six light probes including four indoor environments (Cathedral, Closed Bar, Dining, Grace) and two outdoor environments (Morning, Mountain) for rendering (http://gl.ict.usc.edu/Data/HighResProbes/ and http://illuminatedtools.com/freeprobes/). The right column in Figure 2 shows pure reflective specular objects embedded in each of these light fields. The relative position of camera and light probes was consistent through the study. The camera position was fixed to 10 BU away from the 3D object's center. The light probe images were used as environment maps projected on a sphere of infinite radius in order to illuminate the scene. This rendering setup was used to create movies that were shown to observers, as explained further below in the Procedure. In these movies, the sphere sinusoidally oscillated along the horizontal axis at 0.5 Hz (amplitude = 2 BU, corresponding display size: 9.4 deg) which started from the center and moved rightwards. All the movie sequences were rendered using a custom Python script executed in the Blender 3D environment. All movies were rendered at 60 fps.

In the follow-up experiment, we examined the effect of 3D shape on perceived thickness. We used three different mesh geometries to assess the role of 3D shape (sphere, torus, bumpy; see Figure 4). The sphere was the same as used in the main experiment. The torus was made of an isometric tube whose diameter was 0.2 BU, and the distance from the center of the tube to the center of the torus was 0.4 BU. The angle of the torus was tangential to the camera, thus the total width of the Torus was 1 BU. This setting is a preset geometry of Blender 3D (Add > Mesh > Tours; Major segment = 200, Sub segment = 100, with 1,960,000 triangle faces, 980,000 vertices, subdivisions = 6). The bumpy geometry was the same as the shape used in our previous study (Ohara et al., 2020). In this experiment, the reference matte stimuli had uniform texture instead of the dotted texture, because it was not feasible to apply dot textures to the torus and bumpy shapes. There were four specular ratios of 0, 1, 5, and 40%. There were three light probes used (Morning, Cathedral, and Dining).

### Procedure

Visual stimuli were presented using custom psychophysical software called Psymat (http://juno3d.com/software/) running on a PC (OS: Windows 10 Pro, CPU: Intel Core i7-6700, Graphic card: GeForce GTX 960).

Observers were seated, and their head fixed by a chin rest. Stimuli were presented on an LCD flat-panel display (HP E242) situated 45 cm in front of the observers. The display's gamma was calibrated to 2.2 (mean absolute error was 5.7%, measured by a color meter CS-200, Konica-Minolta). Two stimulus movies were shown, separated horizontally. One of the stimuli was always the matte object, and the other was the test (Supplementary Video 1). Positions of the reference and test were randomized. Each stimulus size was 17 × 17 cm, 21.4 × 21.4 deg, 626 × 626 pixels including the object and background of the light fields, and area other than the stimulus movie was mid-gray (red = green = blue = 128). Observers were given a minute at the start of the experiment to practice a small number of randomly presented trials, before moving on to the formal testing session. The observers saw the stimuli with both eyes open.

It is known that the thickness of matte objects tends to be perceptually underestimated compared to ground truth under a single light source (Mooney and Anderson, 2014). However, thickness perception for diffusely reflecting objects may be more accurate, especially under complex light sources (Wilder et al., 2019). Thus, we reasoned matte objects should be an appropriate reference in complex illumination environments.

After watching the movies, observers were asked to choose which of the 3D objects appeared more elongated (like a rugby ball) in depth using the corresponding arrow key on the standard keyboard. No feedback on their response accuracy was provided. The movie continued to loop seamlessly until the observer responded. There was no time limit for the observer to respond. After pushing the response key, the next movie commenced playing immediately. Order of stimuli was randomized for the entire block of 54 conditions [(1 control + 8 specular ratio) × 6 light fields]. In the control condition, both sides of the display present the matte object rendered within the same light probe. Observers performed 15 repeats for each stimulus condition.

Procedures used in the follow-up experiment were the same as in the main experiment, except for the number of the stimuli [(1 control + 4 specular ratio) × 3 light fields × 15 repetitions].

### Statistical Analysis

The observer's responses were analyzed using a generalized liner model (GLM). All statistical tests were performed using MATLAB (R2020b, Mathworks). The *fitglm* function with the link function for binomial distributions was used to perform GLM analyses. The *coeftest* function was used to calculate the effect of the specular ratio, light probes (in the main experiment), and object shape (in the follow-up experiment).

## Results

**Figure 3:**
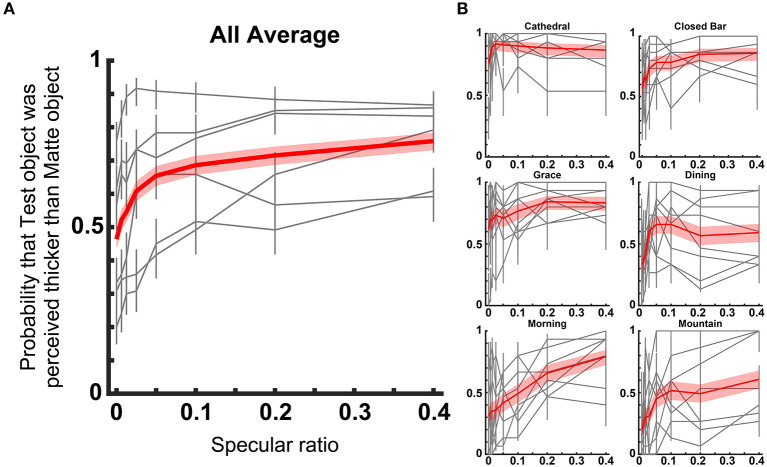
Perceived thickness for different light probes. **(A)** Plots show mean probability that the test object with each specular ratio was perceived thicker than the Matte reference object. The red line shows the average across all light probes and observers. Gray lines show the result for each light probe [shown in **(B)** as a red line]. The red band and gray error bar show the 95% confidence interval. **(B)** Results are shown separately for different light probes. The red line shows the average of all observers. Gray lines show the result of each observer. The red band and gray error bar show the 95% confidential interval.

Perceived thickness of the test objects relative to matte objects are shown in Figure 3. Observed probability increased overall as the specular ratio increased (χ^2^ = 933, *df* = 7, *p* < 0.001, GLM). This pattern in the data was consistent even when the analysis was performed for each light probe separately (Closed Bar: χ^2^ = 32.4, *df* = 2, *p* < 0.001, Grace: χ^2^ = 18.4, *df* = 2, *p* < 0.001, Dining: χ^2^ = 11.7, *df* = 2, *p* < 0.001, Morning: χ^2^ = 102, *df* = 2, *p* < 0.001, Mountain: χ^2^ = 52.8, *df* = 2, *p* < 0.001, GLM). It is important to note that the mean probability estimate across all specular ratios were different (*p* < 0.001, GLM). Across all light probes, the test object simulated with a 40% specular component was perceived thicker than the matte object. In three of six light probes, the Test object of 0% specular component was perceived flatter than the matte object. Hence, the probability estimate of perceived thickness did not increase linearly, but increased sharply around 0–0.05 in specular ratio.

**Figure 4:**
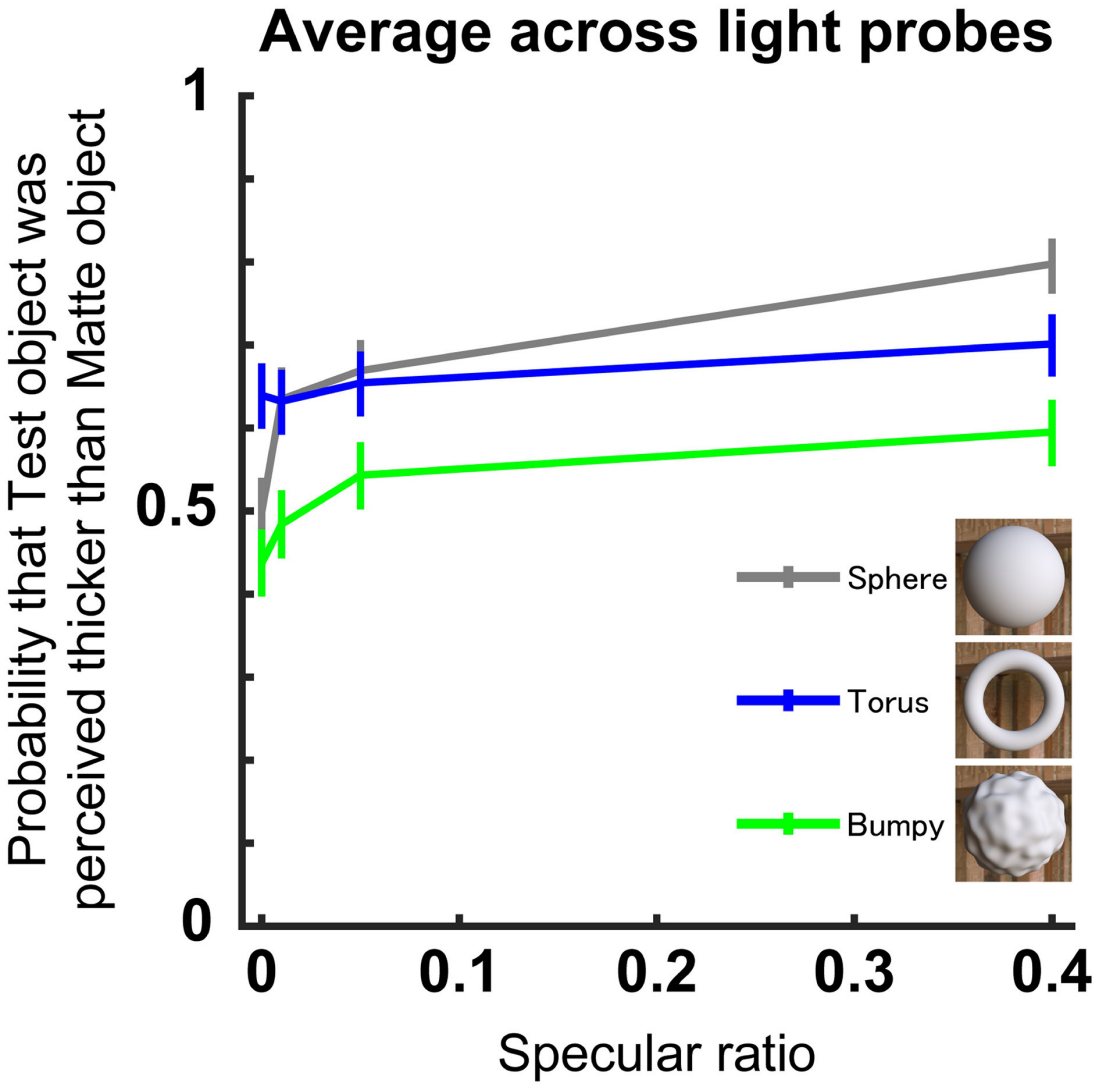
Perceived thickness for different 3D geometry of the object. Format is the same as in Figure 3A. Each line denotes a different 3D geometry, and the averages were computed across the three light probes and observers (*n* = 9). Inset shows the appearance of the three matte shapes illuminated using the Dining light probe.

The effects of different geometric 3D shapes on perceived thickness are shown in Figure 4. Observed response probability increased overall as the specular ratio increased for all three shapes (χ^2^ = 152, *df* = 4, *p* < 0.001, GLM). This pattern in the response data was consistent even when the analysis was performed for each object shape (Sphere: χ^2^ = 62, *df* = 2, *p* < 0.001, Torus: χ^2^ = 5.04, *df* = 2, *p* = 0.02, Bumpy: χ^2^ = 16.4, *df* = 2, *p* < 0.001, GLM). Consistent with the main experiment, there were significant effects of different light probes on perceived thickness (*p* < 0.001, GLM). In summary, this follow-up experiment showed that perceived thickness increased with increasing specular ratio for all 3D shapes, and the increase was prominent around the 0–0.1 specular ratio. However, the torus induced rather consistent perceived thickness judgments irrespective of the specular ratio.

### Point of Subjective Equality

In the main experiment, the test objects that generate 0.5 probability would be perceived to have the ‘true' thickness, assuming the observers perceived the true thickness of the matte object. On average, a specular ratio of 0.049 induced true thickness perception. Hereafter, we use point of subjective equality (PSE) to denote the specular ratio that induces a 0.5 probability estimate of perceived thickness. For three of the six light probes, the PSE could be determined, and they ranged between 0.01 and 0.34 (Dining: 0.01–0.02, Morning: 0.05–0.15, Mountain: 0.05–0.34). The PSE was not determined in the other light probes, as the observed probability was larger than 0.5 at the 0 specular ratio. Thus, the specular ratio at the PSE was overall not large. Those ratios were low suggesting that a small specular component would be enough for unbiased thickness perception. However, there remains a possibility that the effect of the reference stimulus (matte objects) may generate a percept that is different to veridical. This is further compounded by the possibility that the perceived shape of matte objects may also depend on the light probe used. Thus, another index would be useful to determine the critical point of subjective equality in thickness between specular and refractive objects.

### Midpoint

Increasing specular ratio from 0.0 to 0.4 generally increased the probability of perceiving an object as having greater thickness in depth. Which specular ratio generates the mean probability between them? The observed probability estimates increased steeply around the specular ratio from 0.00 to 0.05, but then increased more gradually up to the 0.40 mark. We calculated the specular ratio that induces mean probability in response to the 0 and 40% specular ratios. The midpoint was found to range between 0.02 and 0.2 (Cathedral: 0.02–0.025, Closed Bar: 0.02–0.05, Grace: 0.02–0.145, Dining: 0.02–0.03, Morning: 0.12–0.2, Mountain: 0.024–0.06). For the objects viewed in the three light fields where the PSE could be determined, these two points ranged between 0.01 and 0.34, smaller than the balanced (0.5) specular ratio. The differences in estimated midpoint suggest that different light fields have profound effects on relative judgments of 3D shape when observers compare transparent objects varying in sheen against completely opaque matte objects in the same illumination conditions.

### Image Cues

In a recent study, Chowdhury et al. (2017) assessed whether conventional shape from shading models for matte opaque objects might extend to translucent (i.e., non-opaque) objects. They found that surfaces were judged as bumpier in 3D shape when generating local variations in image luminance over the same finite image space, compared with smoother surfaces. This finding revealed that generic computations of image contrast could be used to infer shape from shading across objects varying in opacity.

Although completely refractive transparent objects lack this diffuse shading, it is possible that similar computations of local image contrast could be used to also infer their 3D shape. Fleming et al. (2004) showed that highly curved regions of specular surfaces generate higher spatial frequency distributions in the contrast variations of the reflected light field. Alternatively, flatter surface regions reduce this spatial frequency, which would have the effect of reducing local contrast energy at local surface regions. Hence, in a similar way to matte objects, we expect that local contrast variations in image structure will still be diagnostic of local surface curvature and thus the thickness of objects in depth. Indeed, previous studies have shown that changes in the distribution of environmental edge contours are informative of an object's material composition and likely also 3D shape (Kawabe et al., 2015; Dövencioglu et al., 2018). Based on this evidence, variations in local image contrast may inform the perception of shape from distortions of the light field transmitted through the body of refractive objects. We explore the utility of computing local RMS contrast for these refractive materials.

**Figure 5:**
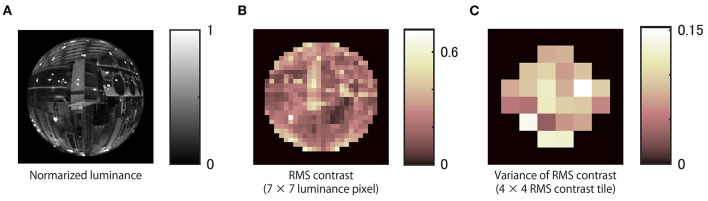
Local RMS contrast variability. **(A)** Normalized luminance image of an example stimulus. **(B)** Local RMS contrasts over finite image regions defined within 7 × 7 pixels were computed. **(C)** The variance of RMS contrast within the regions of 4 × 4 RMS contrast areas was computed. RMS indicates root-mean-square.

It was previously found that the perception of thickness can be modeled by computing variations in local contrast across the object's image (Ohara et al., 2020). Calculating the variance of local root-mean square (RMS) contrast corresponds to the activity of two hierarchical filters. First, local RMS contrast models the activity of the primary visual cortex, which responds to various types of spatial contrast and intensity (Hubel and Wiesel, 1962; Freeman et al., 2013; Rieger et al., 2013). The RMS contrast was preferred for describing detectability of natural images (Bex and Makous, 2002; Pelli and Bex, 2013). Second, the variance of local RMS contrast models the function of cells found at higher levels of visual processing (Fukushima and Miyake, 1982; Freeman et al., 2013). Here, we applied the same model to examine whether perceived object thickness in depth could be explained computationally. The RMS contrast of the stimulus images is computed by the following formula:


$$\begin{array}{l} \text{RMS contrast }=\;\sqrt{\frac{1}{\text{N}}\overset{\text{N}}{\underset{\text{n }=\;1}{\sum }}|{{\text{x}}_{\text{n}}}^{2}|} \end{array}$$

where *x* is pixel luminance after considering gamma correction of the display, and *N* is the number of pixels used to analyse the region of interest. We computed local RMS contrast over finite image regions defined within 7 × 7 pixel square tiles of the luminance image (Figures 5A,B). Then, we calculated the variance of local RMS contrast over regions defined within 4 × 4 tiles (Figure 5C). Thus, each variance was computed over a 28 × 28 pixel region of the stimulus image. This procedure was repeated for all frames of each stimulus movie.

**Figure 6:**
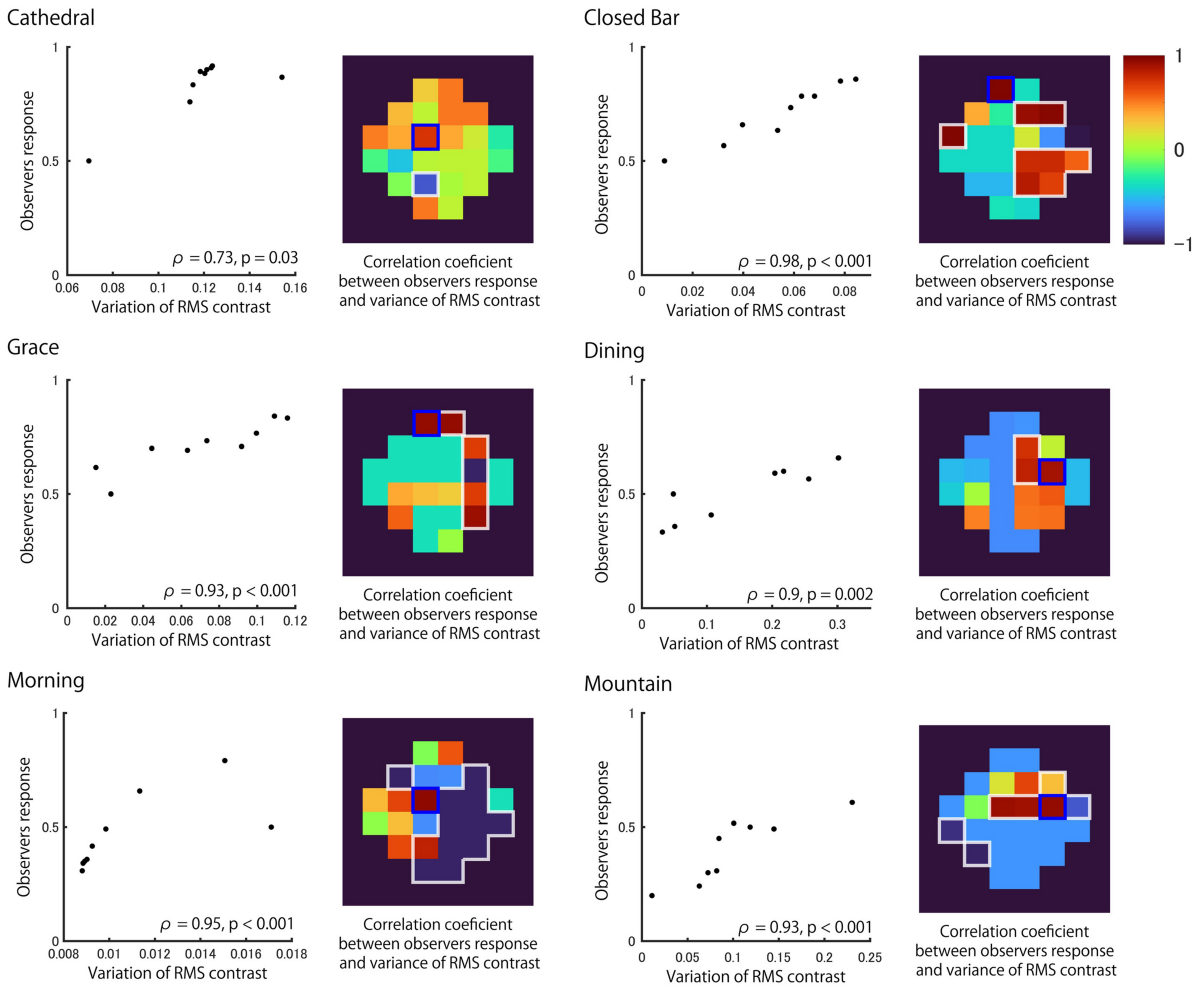
Correlation between local RMS contrast variability and observer responses for each light probe. Correlation plots between the variance in local RMS contrast of the region with highest correlation (denoted by blue square in heatmap) and the observers' response in each light probe. Data includes the reference (matte) stimulus where the corresponding response was assumed to be 0.5. Heatmaps show the Spearman's rank correlation coefficient calculated separately for each region. The region with white outline shows significant correlation (*p* < 0.05).

Figure 6 shows a correlation plot between the variance of local RMS contrasts and the average probability estimate of stimuli being selected as thicker for each light probe. The image regions which have highest positive correlation are designated separately for calculating the variance of local RMS contrast (tiles outlined in blue for each heatmap). The observed highest positive correlation coefficient (Spearman's Rho) was found to be significant (*p* < 0.05). Significant correlations were also observed in other regions of the image (white outline in each heatmap). The highly positive correlations were found in different regions of the surface image across the six light fields. Similar conclusions were found when different regional sizes were used for the calculation of local RMS contrast and the tile size for calculating the variance (Supplementary Figure S2). Correlation coefficients were calculated for each subject separately, showing a similar trend, with significant correlations for all light probes. The above mentioned analysis was performed for a certain frame (center) of the movie, but similar conclusions could be drawn for different stimulus object positions during lateral motion (Supplementary Figure S3). These results indicate thickness perception can be explained by the variance in values of local RMS contrasts, but the surface regions of importance appear to vary across light fields due to differences in the optical structure.

**Figure 7:**
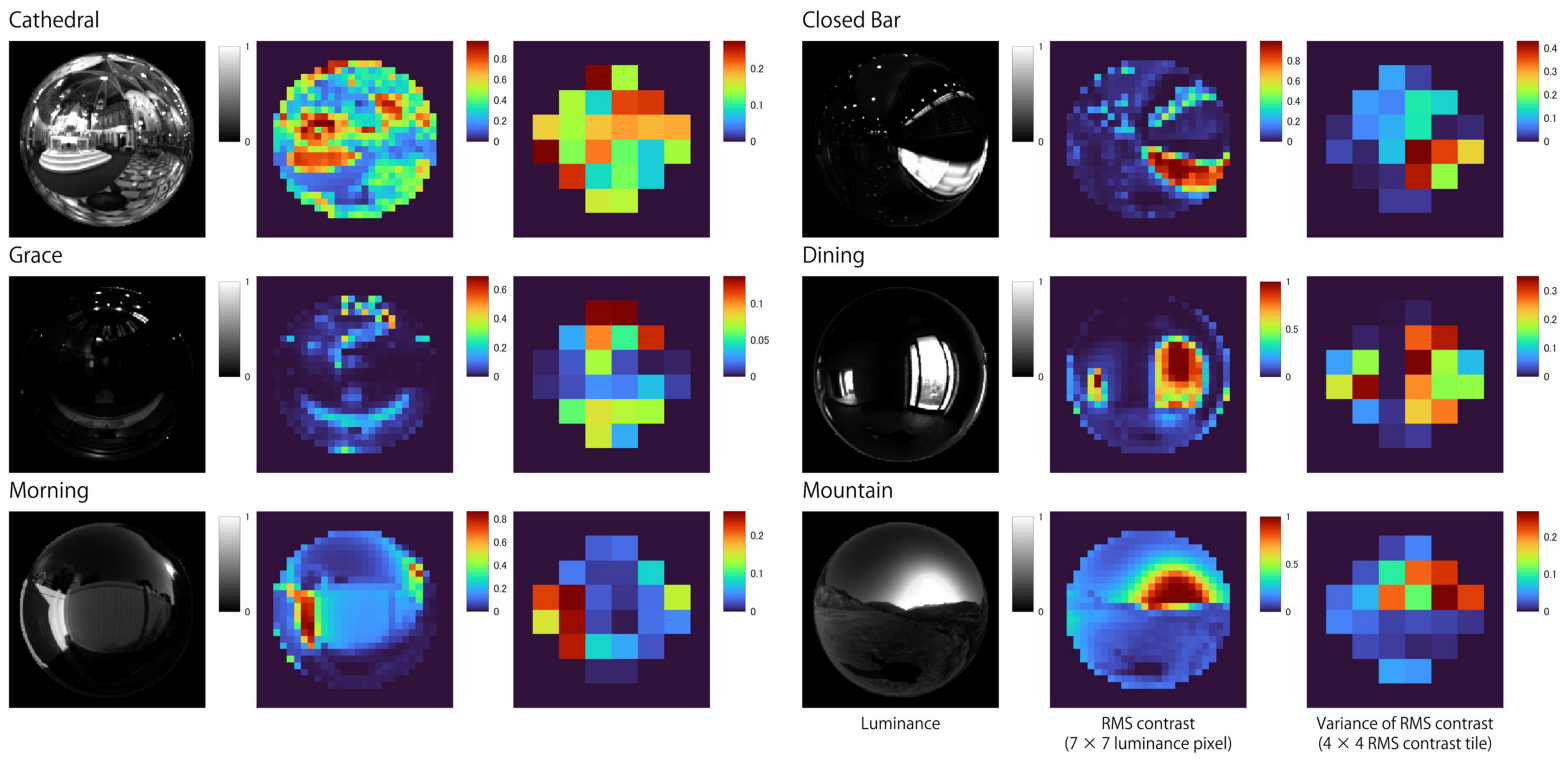
Heatmaps showing the variance in local RMS contrast for the 100% specular surface in each of the six light fields. Note that regions of local maxima for variations in local RMS contrast generally coincide with brighter image regions.

**Figure 8:**
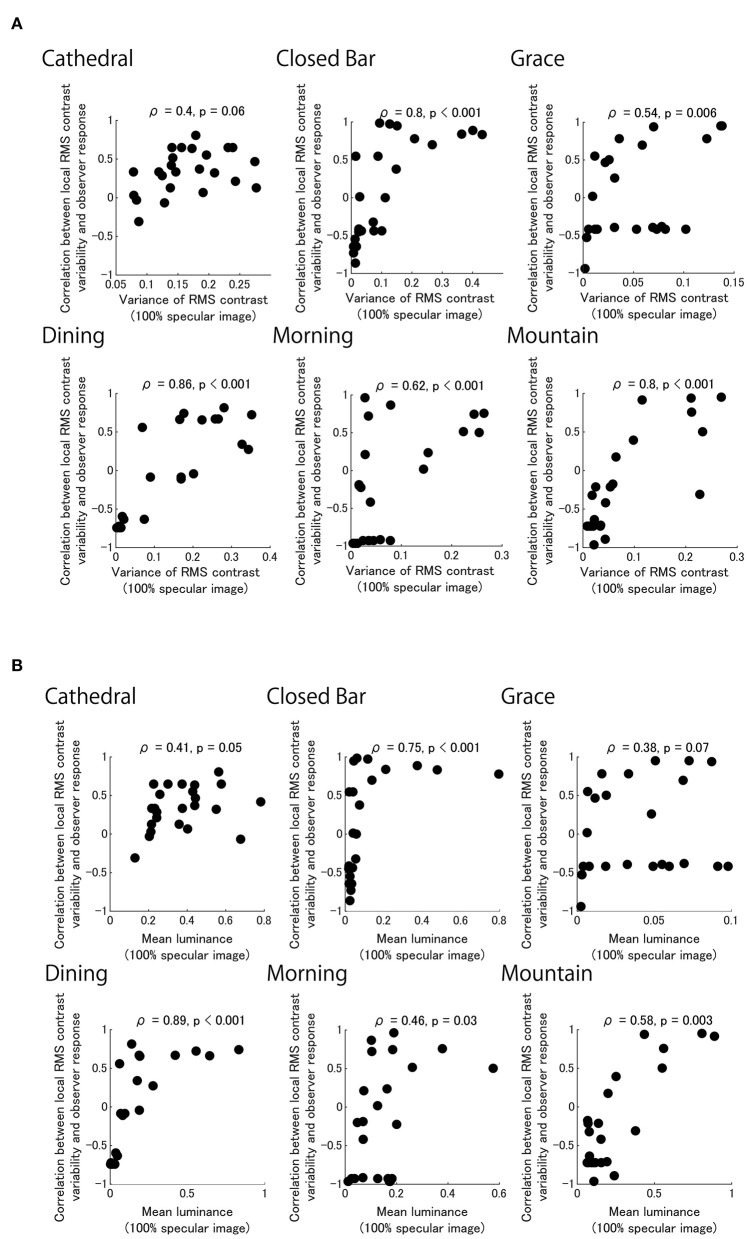
Black dots show each of the 24 correlation coefficients for our model in each pixel region of images shown in Figure 6 plotted as a function of image parameters for the 100% specular image component (Figure 7). Separate panels show model performance for six light fields plotted against variation in local RMS contrast **(A)** and local image luminance **(B)**.

There are tiles that are highly positively correlated for each of the six light probes (Figure 6). The distribution of tiles with high correlation is similar to the distribution of tiles with high variance in the local RMS contrast of the 100% Specular image (Figure 7). This result suggests that the observer may have used the brighter image regions of the reflectance image as a cue to estimate object thickness. Indeed, Figure 8 shows the correlation coefficients between our model (Figure 6) and the 100% specular image's variance in local RMS contrast or mean luminance of the corresponding local neighborhood pixels of each stimulus separately for the six light probes. Generally, image regions of greater luminance and variation in local contrast generated correlations with psychophysical data that were overall stronger, suggesting the visual system may use these local image features to identify critical regions for assessing an object's 3D shape.

The Grace and Cathedral light fields deviate from the pattern seen in the data for the remaining light fields. It is possible that in the case of the very dark Grace light field, image regions that exhibit greater variation in local RMS contrast and not just higher local luminance are used when assessing 3D shape. For the Cathedral light field, the pattern of psychophysical data was confined to a very tight range of very high probability estimates for perceived thickness, which may be attributed to the comparatively very low image contrast generated by the matte reference stimulus.

## Discussion

The experimental results showed that perceived thickness increased as a function of specular ratio—the proportion of the specular component that is combined with the refractive distortion field. In particular, the perceived thickness of objects increased markedly around the specular ratio range of 0–10%. Perceived thickness equivalent to the matte object was observed at <3.4% of the specular ratio. These results are consistent with our previous finding in which the specular objects are perceived as thicker than transparent objects without specular highlights, and the objects with specular highlights and transparency (1:1) are perceived similarly to specular objects (Ohara et al., 2020). Results of the current study indicate that thickness perception changes markedly with very small percentages of specular reflection introduced into the image.

The follow-up experiment showed that perceived thickness increased as a function of specular ratio for all 3D shapes, although the magnitude of this effect varied across the different 3D shapes used. Specifically, we were able to reproduce our finding that perceived thickness increases as a function of specular ratio for the bumpy object, but not for the torus. The reason why the tours shape did not produce similar results to the other shapes may be that it is optically very narrow along its radius in the image. Thus, there was limited spatial variation in the local orientation cues available for the observer to use to form their thickness judgments. This was not the case for the doubly curved spherical, smooth and bumpy objects, presumably due to the rich diversity in local shading cues available.

### Why Does Perceived Thickness Increase Sharply at Low Specular Ratios?

**Figure 9:**
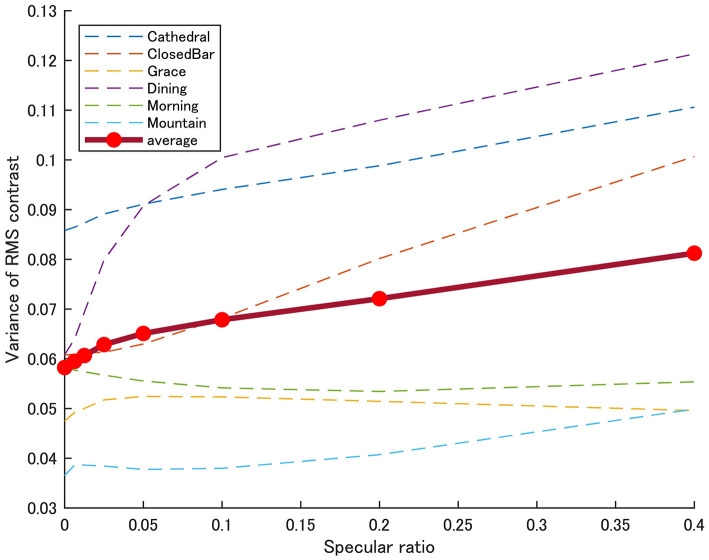
Variance of local RMS contrast by specular ratio. Plots show the mean variance in local RMS contrast of Test objects in all six light probes as a function of specular ratio. The size of the region for calculating the variance of local RMS contrast was the same as used in Figure 5.

Perceived thickness was most steeply increased around the 0–10% specular ratio. One possible explanation for this observation is that human observers are more sensitive to the addition of image features around the point of subjective equality above the matte reference object. Indeed, the observers' response most steeply increased around the point where the 50% probability response was observed, although there were large individual differences and an apparent light-probe dependency. Here, image properties should be more salient around the lowest specular ratio of 0% (i.e., based on Weber's law). We calculated how the variance of local RMS contrast changes with specular ratio (Figure 9). This variance of local RMS contrast image cue is seen to overall vary with specular ratios in a similar pattern to observer responses. The variance of local RMS contrast was highly correlated with the estimates of perceived thickness probabilities (*r* = 0.79, *p* < 0.001). This finding suggests human observers are sensitive to perceiving the shape of natural transparent objects with no specular reflections, or they can also perceive 3D shape in the 100% specular objects. This possibility is discussed in the next session.

### Physical Validity of Correct and Accurate Thickness Perception

The test objects with specular ratios ranging between 0.01 and 0.34 were perceived as having similar thickness to the matte-textured 3D models' thickness. What visual cue might best help explain this result? There are several possible reasons that we explore below.

First, most ordinary objects have <5% specular reflectance. For example, crown glass has 4.2% specular reflectance, and ice has 2% in air (Polyanskiy, 2008). It is possible that the participants were ecologically familiar with transparent objects through daily life learning and interaction. They would have been able to correctly estimate the thickness of the test stimulus when its reflectance was thus in the 3–5% range. They were also sensitive to the addition of contrast variations generated by the addition of small reflectance levels, thus the observed psychometric function was steep around these specular ratios.

Secondly, some artifacts may serve as cues because we used physically less rigorous rendering for transparent objects. In the rendering, we used a specific refractive index (1.51) to simulate transparent objects irrespective of the specular ratio. The image based on a specific refractive index (1.51) is perceived equivalent to the matte surface when the specular ratio is 4.3%, but when the reflectance deviates from this value, the image might look unnatural. Because it is known that reflectance (*R*) of an object is uniquely determined by the refractive index (*n*) (Hecht, 2001).


$$\begin{array}{l} \text{R }=\;{\left(\frac{1\;-\text{ n}}{1\;+\text{ n}}\right)}^{2} \end{array}$$

From the equation, the transparent object which has 1.51 refractive index reflects 4.3% of light when the incident light comes from the normal direction. However, RI = 1.3 generates 1.7% reflection, and RI = 2.0 generates 11% reflection. According to this view, the observer was able to estimate the correct thickness of the test stimulus around 4.3% of the specular ratio based on the transparent image with 1.51 RI. However, this possibility is unlikely because one study has shown that observers are unable to estimate the refractive index accurately (Fleming et al., 2011). Nevertheless, observers may be familiar with the natural RI of common substances like water and glass. It is possible therefore that observer judgments of thickness may depend on familiarity with certain RIs and the image cues they generate.

### Unnatural Stimuli

**Figure 10:**
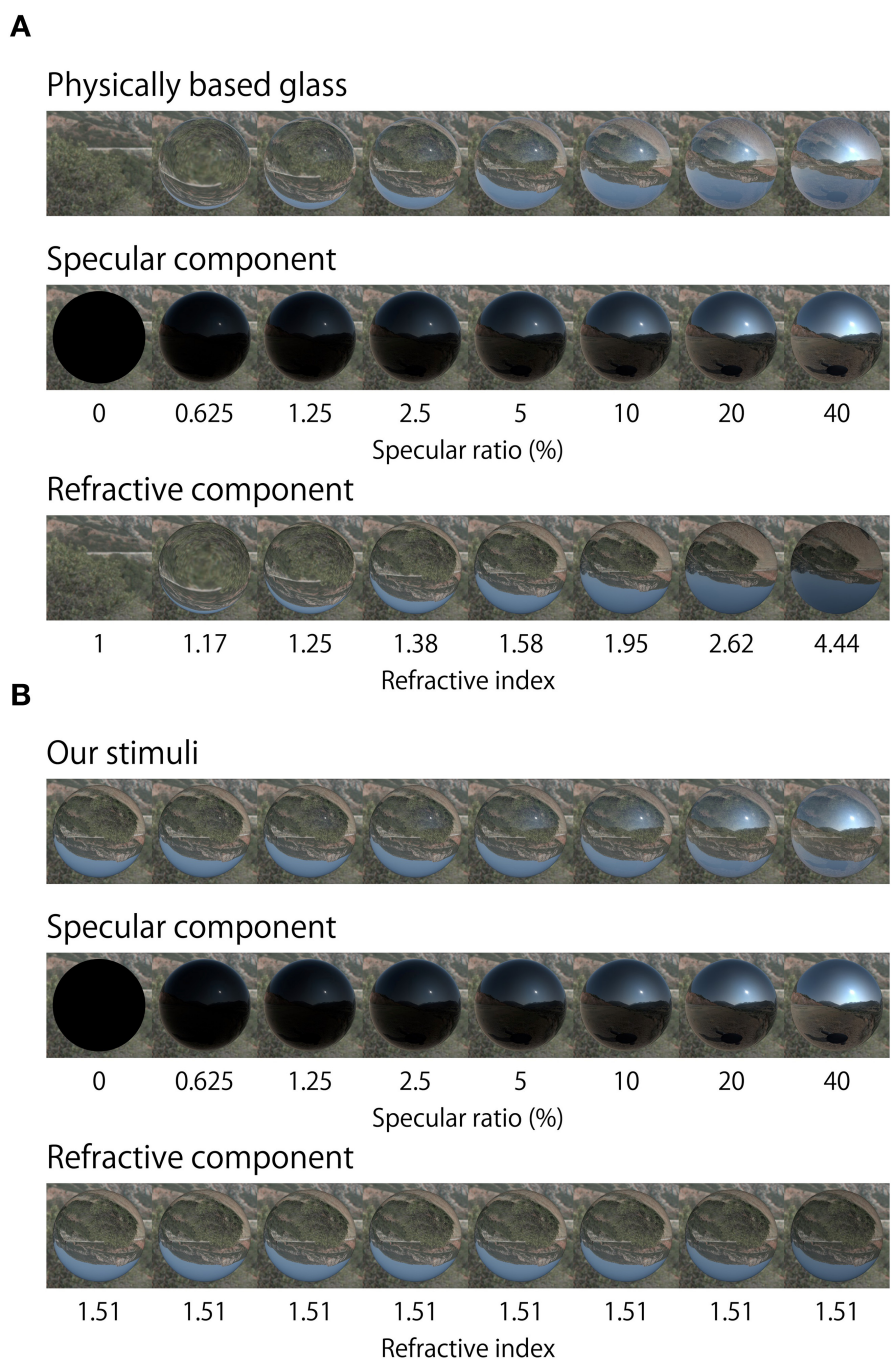
Physically based rendering and our stimuli renderings. **(A)** Physically based rendering changes object refractive distortion along with specular ratio. Middle and bottom rows show the specular and refractive components of the image on the top row. Refractive images were rendered by variable refractive index. **(B)** Top row shows objects which were rendered using the method of our experimental stimuli. Middle and bottom rows show the specular and refractive components of the image in the top row. Refractive images were rendered using a fixed refractive index.

The upper row in Figure 10A shows physically more rigorous rendering of transparent objects with various refractive indexes. Images in the middle and bottom rows show specular and refractive components of the image in the top row. We note that not only relative intensity of specular and refractive components vary, but also the transparent image deforms depending on the RI. In contrast, in our stimulus, image deformations of transparent objects were the same across specular ratios because we used a fixed RI (1.51) (Figure 10B). We only manipulated relative intensity of specular and refractive components to examine the effect of specular ratio.

The reason why we adopted this rendering approach is that we aimed to examine the relative contributions of the intensity of the specular reflection while keeping the deformation of the refractive image constant. Image distortion may affect thickness perception, nevertheless our simplification of the rendering manipulation reveals the importance of relative specular amplitude rather than the effect of image distortion *per se*. This approach is different from previous studies that examined the relationship between shape perception and refractive index estimated by changing the degree of distortion (and intensity of specular reflections) generated by manipulating refractive index (Fleming et al., 2011; Kawabe and Kogovšek, 2017; Schlüter and Faul, 2019).

We have already found that the refractive component reduced thickness perception (Ohara et al., 2020). In contrast, the reflective component enhances thickness perception (Nishida and Shinya, 1998; Mooney and Anderson, 2014). To understand the relative contribution of the specular and refractive components, it was necessary to manipulate the specular ratio in combination with image structure attributed to other material properties like transparency.

The image processing required to compute variance in local RMS contrast involves calculations from luminance values directly obtained from the images with both reflective and refractive components combined, rather than calculating it based on the reflective and refractive images separately. We have already found that mixing the two images by 50% generates the same thickness perception as achieved by the specular image alone (Ohara et al., 2020), and since the two images are different, the variance of local RMS contrast will produce different outputs. It is still possible that observers may have estimated the object's thickness after first separating specular and transparent components, rather than directly from the combined image.

The use of unnatural stimuli may have caused the subjects to perceive the spherical objects as bubbles instead of solid transparent objects, or non-rigid shape changes with the objects' movement (Kawabe et al., 2015; Dövencioglu et al., 2018). Although we did not ask the observers to report their perceptual estimates of material class or rigidity in the experiment, none of the participants reported these distortions.

### Effect of Light Probe

The perceived thickness of objects varied depending on the light probe used. In some cases, response probabilities were consistently high (i.e., Cathedral and Closed bar), and in some other cases, the probabilities were relatively low and showed continuous increase (i.e., Morning and Mountain). In our previous experiment, different light probes were used (Grove and St Peters), and similar conclusions were obtained (that the pure specular image induced overestimation and the pure refractive image induced relative underestimation). It is also known that the structure of the surrounding environment affects the appearance of the refractive object (Nefs et al., 2006; Khang et al., 2007; Fleming et al., 2009; Chowdhury et al., 2017). The divergence could be due to the difference in image-based features such as variance of the local RMS contrast and other image properties (Zhang et al., 2019).

### The Generality of Variance of Local RMS Contrast Model

We analyzed observers' responses based on variance of local RMS contrast in stimulus images including matte, specular, and transparent objects. The motivation for using variance of local RMS contrast came from previous work on the perceived shape from shading of translucent objects (Chowdhury et al., 2017). Indeed, other previous works have suggested that the visual system relies on common image features, irrespective of their material differences. For instance, humans appear to use common image features when perceiving the shape of matte and velvet objects (Wijntjes et al., 2012; Sawayama and Nishida, 2018). Some studies have attempted to devise generic models for the perceived shape of opaque objects (Fleming et al., 2004, 2011; Kunsberg and Zucker, 2021). However, neurophysiological research further supports the view that there are generic mechanisms of 3D shape recovery in the brain. A recent neuroimaging study has reported that there are V4 neurons selective to shape information irrespective of material differences (Srinath et al., 2021). Together, these studies indicate there exist potentially generic shape cues that are independent of an object's material properties. Although these cues may be context specific, one possible cue might be the variance of local RMS contrast proposed here, which was found to be applicable to the estimation of 3D shape of objects with different simulated material compositions.

## Conclusion

We explored the visual perception of shape in computer-generated objects with different surface optics. Our results suggest that the specular component serves as a depth cue after providing up to a 5% contribution to the final image, consistent with the natural optical interaction of light with smooth refractive objects. Excessive specular ratio produced excessive object thickness perception compared to natural transparent objects, suggesting the human visual system is tuned to correctly recognize the shapes of solid transparent objects as they have specific specular ratios. Conversely, if there are no specular reflections, the thickness of the object is perceptually underestimated. We were able to show that increasing specular ratio increased the perceived thickness of the object, possibly overriding the underestimation in the thickness of objects rendered solely with transparent properties. It would be advantageous to determine how the perceptual effects of specular ratio might hold relevant when absolute estimates of shape are obtained through rating scales or matching tasks. Hopefully, the findings reported here will inform developments in these future studies.

## Data Availability Statement

The raw data supporting the conclusions of this article will be made available by the authors, without undue reservation.

## Ethics Statement

The studies involving human participants were reviewed and approved by Toyohashi University of Technology Ethics Committee. The patients/participants provided their written informed consent to participate in this study.

## Author Contributions

MO conducted the experiments and analyzed the results. All authors conceived the experiments and wrote the article. All authors contributed to the article and approved the submitted version.

## Funding

This study was supported by Leading Graduate School Program R03 of MEXT to MO, Australian Research Council (ARC) Future Fellowship to JK (FT140100535), and JSPS KAKENHI Grant Numbers JP15H05917, JP20K12022, JP20H00614, JP19K22881, and JP21H05820 to KK. This work was supported in part by the Sensory Processes Innovation Network (SPINet).

## Conflict of Interest

The authors declare that the research was conducted in the absence of any commercial or financial relationships that could be construed as a potential conflict of interest.

## Publisher's Note

All claims expressed in this article are solely those of the authors and do not necessarily represent those of their affiliated organizations, or those of the publisher, the editors and the reviewers. Any product that may be evaluated in this article, or claim that may be made by its manufacturer, is not guaranteed or endorsed by the publisher.
