# Supplementary material for: The Role of Specular Reflections and Illumination in the Perception of Thickness in Solid Transparent Objects
Source: Front Psychol. 2022 Feb 17;13:766056. doi: 10.3389/fpsyg.2022.766056 (PMC8891632; doi:10.3389/fpsyg.2022.766056)
Supplement: Supplementary file 2 [file Data_Sheet_1.docx]

Supplementary Material for

The role of specular reflections and illumination in the perception of thickness in solid transparent objects

Masakazu Ohara, Juno Kim, Kowa Koida

**
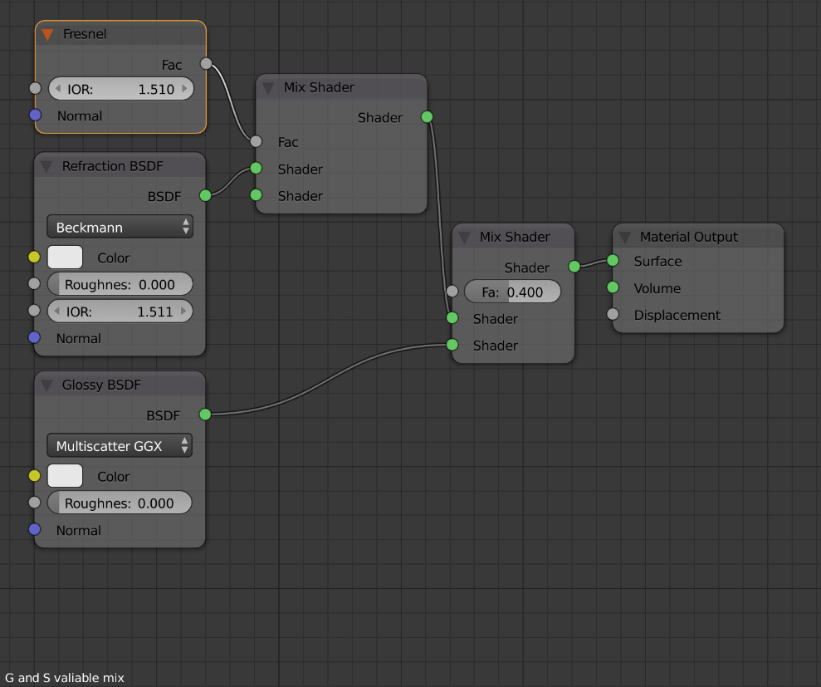
**

**Supplementary Figure 1.** Blender node setup in the Test surface.

**
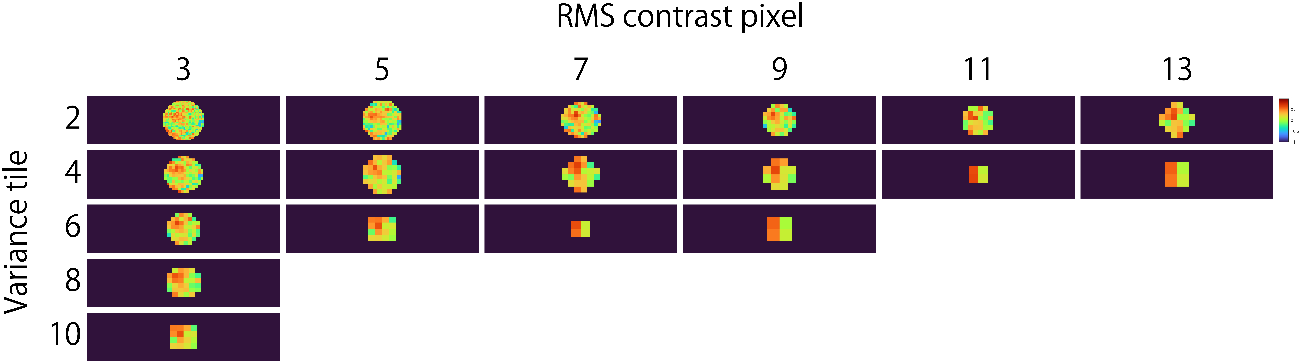
**

**Supplementary Figure 2.** Correlation between various calculation range of local RMS contrast variability and observer response. Heatmap shows the correlation between local RMS contrast variability and experimental result of all six light probes. Horizontal number shows calculation range of local RMS contrast. Vertical number shows calculation range of variance.


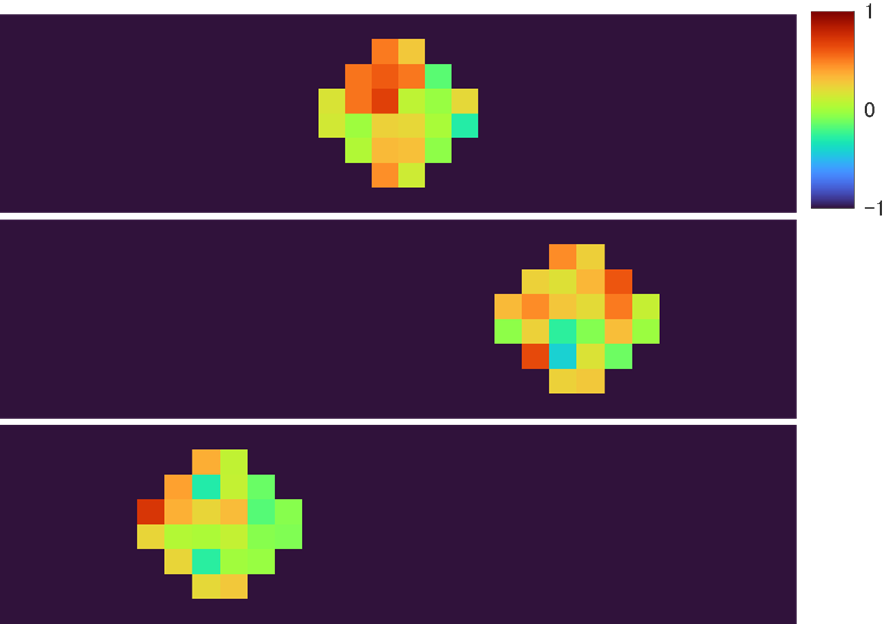


**Supplementary Figure 3.** Correlation between various calculation range of local RMS contrast variability and observer response in each object position. Heatmap shows the correlation between local RMS contrast variability and experimental result of average of all six light probes. The position of the object shows center, right end and left end of the oscillating motion.
